# Abnormalities in substance P neurokinin-1 receptor binding in key brainstem nuclei in sudden infant death syndrome related to prematurity and sex

**DOI:** 10.1371/journal.pone.0184958

**Published:** 2017-09-20

**Authors:** Fiona M. Bright, Robert Vink, Roger W. Byard, Jhodie R. Duncan, Henry F. Krous, David S. Paterson

**Affiliations:** 1 Discipline of Anatomy and Pathology, Adelaide Medical School, University of Adelaide, Adelaide, SA, Australia; 2 Department of Pathology, Boston Children’s Hospital and Harvard Medical School, Boston, MA, United States of America; 3 Sansom Institute for Health Research, University of South Australia, Adelaide, SA, Australia; 4 Florey Institute of Neuroscience and Mental Health, University of Melbourne, Parkville, VIC, Australia; 5 Department of Pathology, Children’s Hospital-San Diego, San Diego, CA, United States of America; Pennsylvania State University, UNITED STATES

## Abstract

Sudden infant death syndrome (SIDS) involves failure of arousal to potentially life threatening events, including hypoxia, during sleep. While neuronal dysfunction and abnormalities in neurotransmitter systems within the medulla oblongata have been implicated, the specific pathways associated with autonomic and cardiorespiratory failure are unknown. The neuropeptide substance P (SP) and its tachykinin neurokinin-1 receptor (NK1R) have been shown to play an integral role in the modulation of homeostatic function in the medulla, including regulation of respiratory rhythm generation, integration of cardiovascular control, and modulation of the baroreceptor reflex and mediation of the chemoreceptor reflex in response to hypoxia. Abnormalities in SP neurotransmission may therefore result in autonomic dysfunction during sleep and contribute to SIDS deaths. [^125^I] Bolton Hunter SP autoradiography was used to map the distribution and density of the SP, NK1R to 13 specific nuclei intimately related to cardiorespiratory function and autonomic control in the human infant medulla of 55 SIDS and 21 control (non-SIDS) infants. Compared to controls, SIDS cases exhibited a differential, abnormal developmental profile of the SP/NK1R system in the medulla. Furthermore the study revealed significantly decreased NK1R binding within key medullary nuclei in SIDS cases, principally in the nucleus tractus solitarii (NTS) and all three subdivisions of the inferior portion of the olivo-cerebellar complex; the principal inferior olivary complex (PIO), medial accessory olive (MAO) and dorsal accessory olive (DAO). Altered NK1R binding was significantly influenced by prematurity and male sex, which may explain the increased risk of SIDS in premature and male infants. Abnormal NK1R binding in these medullary nuclei may contribute to the defective interaction of critical medullary mechanisms with cerebellar sites, resulting in an inability of a SIDS infant to illicit appropriate respiratory and motor responses to life threatening challenges during sleep. These observations support the concept that abnormalities in a multi-neurotransmitter network within key nuclei of the medullary homeostatic system may underlie the pathogenesis of a subset of SIDS cases.

## Introduction

SIDS is a devastating and unexpected event in which a seemingly healthy infant dies in the first year of life during a sleep period, with no warning or prior indication of any adverse pathology to cause alarm [1]. While the precise cause of death in SIDS has not been identified multiple neuropathologic studies have provided evidence that a certain subset of SIDS infants are not entirely 'normal' prior to death [2–5]. Instead these infants possess some form of underlying vulnerability exposing them to an increased risk for sudden death [1, 5, 6]. It is thought that SIDS or a certain subset of SIDS is caused by some form of underlying neural or systematic abnormality in medullary homeostatic control that impairs critical responses to life-threatening challenges such as hypoxia during a sleep period [1]. This failure is thought to result from abnormalities in a multi-neurotransmitter network of neural pathways in the medulla oblongata that control respiration, chemosensitivity, autonomic function and arousal. Indeed abnormalities in various brainstem neurochemicals including catecholaminergic, nicotinic, muscarinic, cholinergic, glutamatergic and neuropeptide systems have been reported [7–11]. Abnormalities in the medullary serotonergic (5-Hydroxytryptamine [5-HT]) system have been the most significantly and consistently observed in the brainstem of SIDS infants, however it remains unclear whether these abnormalities are the primary event in SIDS or an epiphenomenon, with the underlying pathogenesis of these specific abnormalities still undetermined. Furthermore, it is unlikely that dysfunction in only one neurotransmitter system exists given that the actions of neurochemicals are determined by the concurrent modulation and interaction with one another and any deficiencies in one will be immediately compensated by the action of others [12, 13].

The neuropeptide SP has been shown to play an integral role in the modulation of homeostatic function in the medulla, in conjunction with other neurochemicals such as 5-HT, including regulation of respiratory rhythm generation [14–16], integration of cardiovascular control [17], modulation of the baroreceptor reflex [18] and mediation of the chemoreceptor reflex in response to hypoxia [19, 20]. Abnormalities in SP neurotransmission may play, therefore, a role in homeostatic dysfunction in conjunction with other neurotransmitter network deficits in SIDS. Previous studies analyzing SP and NK1R in the brainstem in SIDS have however been inconsistent and inconclusive. Therefore the present study used [^125^I] Bolton Hunter substance P ([^125^I] BH-SP) autoradiography to map the distribution and binding density of the SP, NK1R to 13 specific nuclei intimately related to cardiorespiratory function and autonomic control within the medulla of SIDS cases compared to non-SIDS controls. The medullary nuclei selected for analysis have previously been implicated in the pathogenesis of SIDS with abnormalities in other neurotransmitter systems identified[21–24]. For the first time this study provides evidence for a significant abnormality in SP neurotransmission within key medullary nuclei in SIDS and further supports a role for abnormalities in a multi-neurotransmitter network thought to underlie the pathogenesis of a subset of SIDS infants.

## Materials and methods

### Clinical database

Fresh frozen human infant medullae of 76 infants were sourced from the Office of the Chief Medical examiner in San Diego over the period of 2004 to 2015. The study protocol was approved by the committee on clinical investigation at the Children’s Hospital Boston, MA, USA. The cohort comprised of 41 male and 35 female infants. All cases had a post mortem interval (PMI) less than 30 hours. Deaths were classified as SIDS (n = 55), acutely ill controls (n = 15), chronically ill controls (n = 4) and hypoxic controls (n = 2). SIDS cases were classified according to the San Diego definition [25]. Acute control cases were defined as infants who died acutely and in whom a definitive cause of death was established, chronic controls were defined as an infant under 1 year of age with a history of chronic or repetitive hypoxemia associated with underlying cardiac, pulmonary, or neurological disorder and hypoxic controls were diagnosed according to definitive pathological findings at autopsy. Brainstems did not demonstrate pathologic changes and were all histologically normal.

### Assessment of [^125^I] BH-SP binding to NK1R in human infant medulla

**Fig 1:**
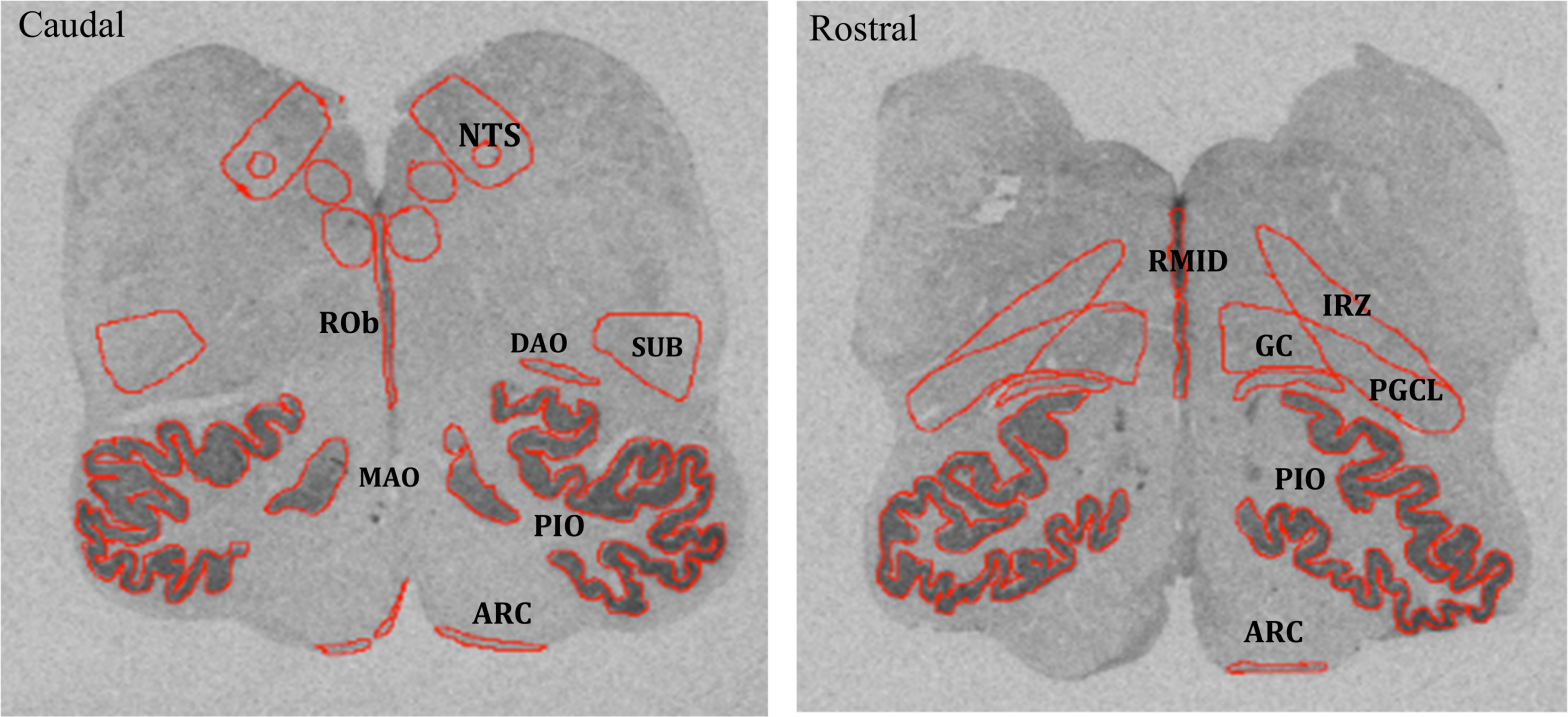
Autoradiographic grey scale images of ^125^I Bolton Hunter SP binding to NK1R receptors in transverse sections of the caudal and rostral human infant medulla. Red boundary contour delineates the 13 target nuclei.

Fig 1 displays Autoradiographic grey scale images of [^125^I] BH-SP binding to NK1R in transverse sections of the caudal and rostral human infant medulla. 13 nuclei in total of the human infant medulla in each specimen were targeted for analysis at both the caudal and rostral medullary levels. The caudal-mid medulla constituted the level of the nucleus of Roller (Plate X), and the rostral medulla (Plate XII) according to the atlas of Olszewski [26]. Nuclei included the raphe obscurus (ROb), midline raphe (RMid), nucleus of the solitary tract (NTS), dorsal motor nucleus of the vagus (DMX), hypoglossal nucleus (HG), intermediate reticular zone (IRZ), gigantocellularis nucleus (GC), paragigantocellularis lateralis nucleus (PGCL), dorsal accessory olive (DAO), principle inferior olive (PIO), medial accessory olive (MAO), subtrigeminal nucleus (SUB) and arcuate nucleus (Arc) at the above defined levels of the brainstem according to the atlas of Olszewski and Baxter (1954). The raphe nuclei were classified according to Tork and Hornung (1990).

Determination of SP receptor specific binding density was performed using 0.15nM [^125^I] Bolton Hunter labeled Lys3 substance P autoradiography and expressed as the specific activity of tissue-bound ligand based on a previously reported protocol [27]. Unfixed brainstems were stored frozen at -80°C and subsequently sectioned at 20μm on a Leitz cryostat and thaw mounted onto super frost plus, glass microscope slides (Thermo Fisher Scientific). Receptor binding density expressed as the specific activity of tissue-bound ligand was analyzed. SP specific binding density was performed using 0.15nM [^125^I]- BH-SP autoradiography. Sections were pre-incubated in 50nM TrisHCl (pH 7.4), 0.02% bovine serum albumin for 15 minutes at room temperature, and then incubated in the same buffer containing 0.1nM [^125^I] BH-SP (NEX190, Perkin-Elmer Inc., Wellesley MA, USA), 3nM MnCl_2,_ Chymostatin 2ug/ml, Leupeptin 4μg/ml and Bacitracin 40μg/ml for 60 minutes at room temperature. Nonspecific binding was determined in the presence of 5μm SP added to the incubation solution. Sections were then washed 1x 2 minutes at room temperature in 50nM Tris-HCl (pH 7.4), 6x1 minute in ice cold 50nM Tris-HCl (pH 7.4) and finally 1x 2 minutes at room temperature in distilled H_2_0 + 0.02% BSA. Sections were then dried in warm air before being placed in cassettes and exposed to ^125^I-sensitive film (Kodak BMR) for 10 days along with a set of ^125^I standards (Amersham) for conversion of optical density of silver grains to fmol/mg tissue.

Film autoradiograms were generated according to standard laboratory procedure for development of light-sensitive film. Digital autoradiogram images of SP specific receptor binding in target nuclei of the human infant medulla were generated as TIFF files from the autoradiography film using MCID Imaging system (Imaging Research, Ontario, Canada). Autoradiograms were generated in grey scale prior to using MCID software to calibrate the images to ^125^I radioactive standards for normalization. Quantitative densitometry analysis of total and non-specific binding density was then measured in fmol/mg in the 14 specific nuclei of interest (all 14 nuclei were not available in all cases). Total receptor binding was determined in 2 sections (2 autoradiograms for each nucleus) and non-specific receptor binding in 1 section for each nucleus analyzed. Specific receptor binding density was determined by subtracting nonspecific binding from total binding.

### Statistical analysis

Statistical analysis of covariance (ANCOVA) was performed to model the difference between NK1R binding at each nuclei and various combinations of diagnosis, controlling for parameters including postnatal age (PNA), postconceptional age (PCA), sex, prematurity status and post mortem interval (PMI). T-tests were used to compare PCA between SIDS cases and controls. Differences were considered significant at p < 0.05.

## Results

### Clinicopathological data

Table 1 summarizes the Clinicopathological data of the study cohort. There were a total of 19 premature cases in the cohort ranging from 26 to 69.29 PCA weeks with a median GA of 29.51 weeks. Non-premature cases ranged from 36.3 PCA weeks (term birth) to 76 PCA weeks. Median PMI for the entire cohort was 19.15 hours, with a range of 0.5–30 hours. PMI had no significant effect on NK1R binding in any of the nuclei analyzed in either SIDS cases or non-SIDS controls and there were no significant differences in PMI between SIDS and controls (p = 0.244). Similarly, no significant differences in NK1R binding were observed between acute, chronic and hypoxic control groups, therefore it was appropriate to combine these groups into a combined control cohort for subsequent analysis.

**Table 1:** Clinicopathological data.

	SIDS (n = 55)	Acute (n = 15)	Combined controls* (n = 21)
**Age mean**			
**PCA (±SD) weeks**	52.11 (7.88)	42.45 (10.27)	44.98 (11.73)
**GA (±SD) weeks**	37.17 (4.82)	35.4 (5.3)	36 (4.72)
**PNA (±SD) weeks**	21.86 (34.28)	10.48 (11.78)	11.43 (12.2)
**Term birth**	43	13	18
**Premature birth**	12	2	3
**PMI mean (±SD) hours**	19 (5.73)	16.82 (7.4)	17.62 (6.64)
**Male**	33	8	12
**Female**	22	7	9

*acute, chronic and hypoxic controls

### [^125^I] BH-SP binding to NK1R in human infant medulla in SIDS vs. controls

**Fig 2:**
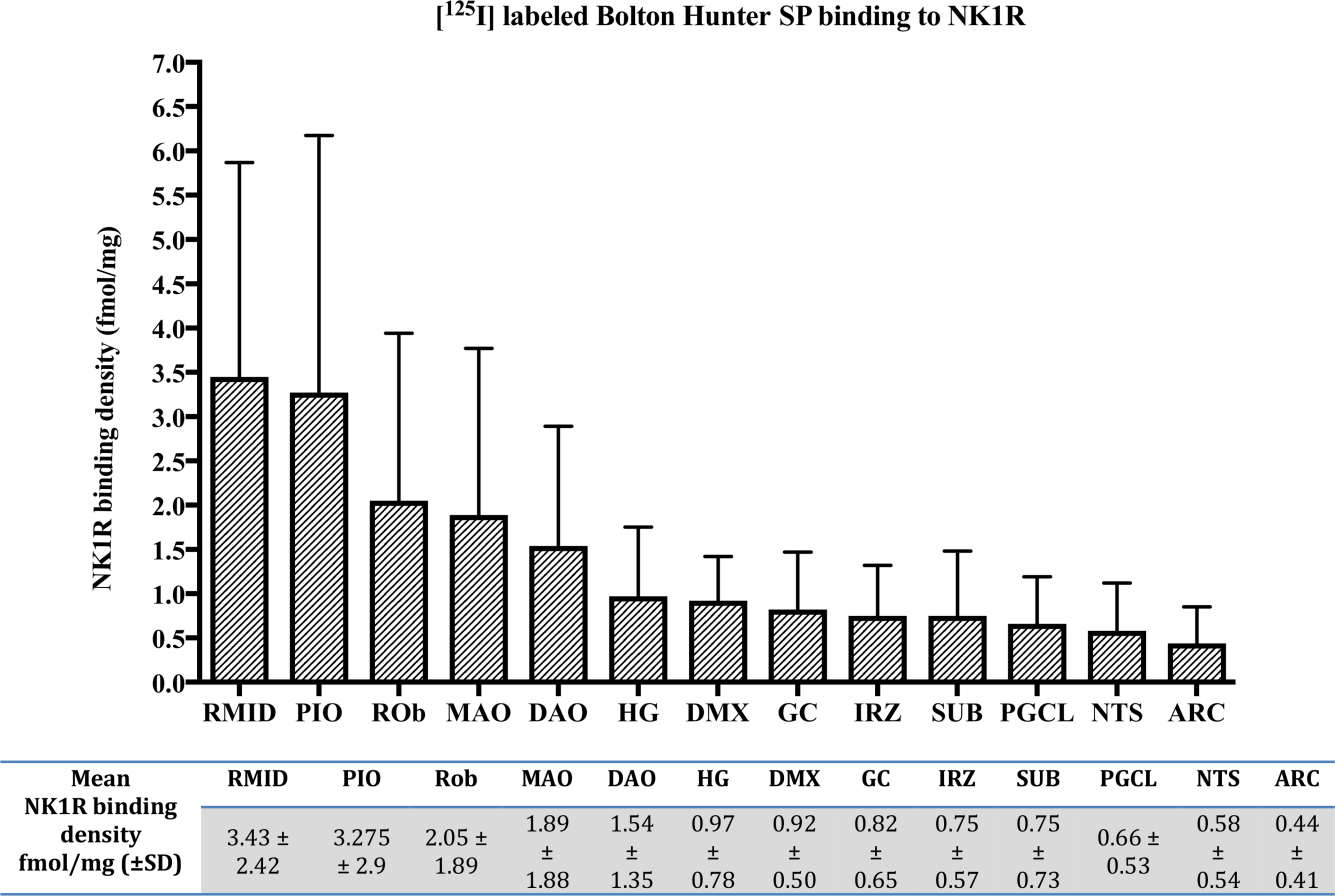
Normative distribution and density of mean total NK1R binding (fmol/mg) in the human infant medulla of non-SIDS controls.

Fig 2 shows the normative distribution and density of mean total NK1R binding in the human infant medulla of non-SIDS controls. The highest density of NK1R binding (>2 fmol/mg) in the medulla of the normal human infant (non-SIDS controls) was observed in the RMid nuclei and PIO nuclei. High binding (>1 fmol/mg) was also present in the ROb, MAO and DAO, while intermediate to low binding (<1 fmol/mg) was present in the HG, DMX, GC, IRZ, SUB, PGCL and NTS. Very low to negligible binding (<0.5 fmol/mg) was present in the ARC (Fig 2) and was measureable in an insufficient number of cases to allow appropriate statistical analysis; therefore we excluded the ARC nuclei from further analysis in the study.

**Fig 3:**
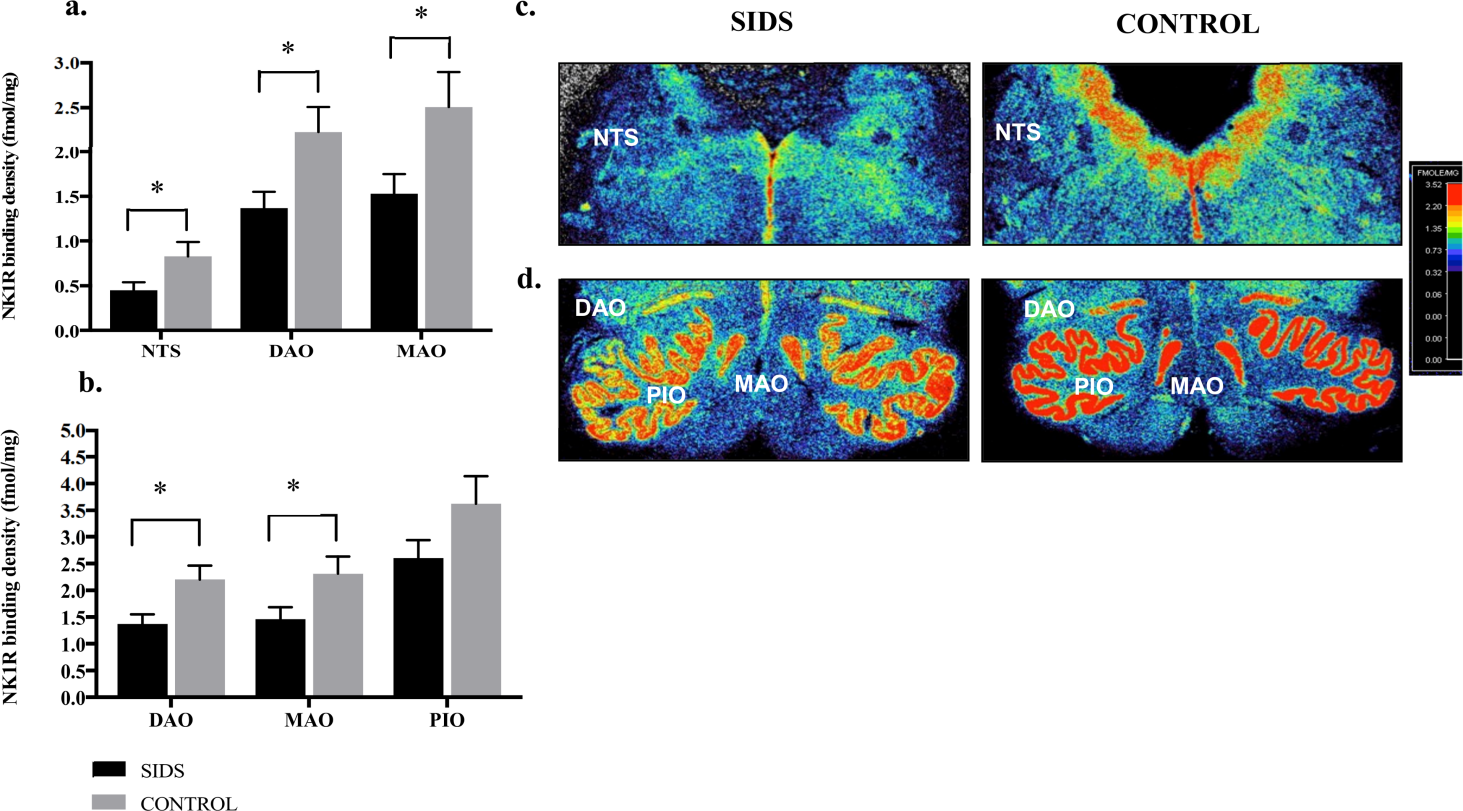
Consistent significant absolute reductions in NK1R binding (fmol/mg) in SIDS cases compared to non-SIDS controls. **a.** NK1R binding SIDS vs. acute controls. Compared to acute controls, NK1R binding was significantly reduced in SIDS cases in the NTS (p = 0.04), DAO (p = 0.01) and MAO (p = 0.03), **b.** NK1R binding in SIDS vs. combined controls. NK1R binding was significantly reduced in SIDS cases in the DAO (p = 0.01) and MAO (p = 0.03) with borderline significance in the PIO (p = 0.09) when compared to all controls combined (acute, chronic and hypoxic).**c.** Autoradiograms displaying NK1R binding (fmol/mg) in the NTS nuclei. Absolute reductions in NK1R binding were consistently observed in SIDS cases. **d.** Autoradiograms displaying NK1R binding (fmol/mg) in component nuclei of the IO. NK1R binding was consistently reduced in SIDS cases. *p = <0.05, **p = <0.01.

Fig 3 shows consistent significant absolute reductions in NK1R binding (fmol/mg) in SIDS cases compared to controls using an ANCOVA model adjusting for PCA. Compared to acute controls, NK1R binding was significantly reduced in SIDS cases in the NTS (p = 0.04), DAO (p = 0.01) and MAO (p = 0.03) (Fig 3A) and significantly reduced in the DAO (p = 0.01) and MAO (p = 0.03) with borderline significance in the PIO (p = 0.09) when compared to all controls combined (acute, chronic and hypoxic) (Fig 3B). Fig 3 also shows autoradiograms displaying NK1R binding in the NTS and component nuclei of the IO within the medulla in a SIDS versus control case, with absolute reductions in NK1R binding consistently observed in SIDS cases within these nuclei (Fig 3C and 3D).

### Analysis of [^125^I] BH-SP binding to NK1R adjusted for age and prematurity status

**Fig 4:**
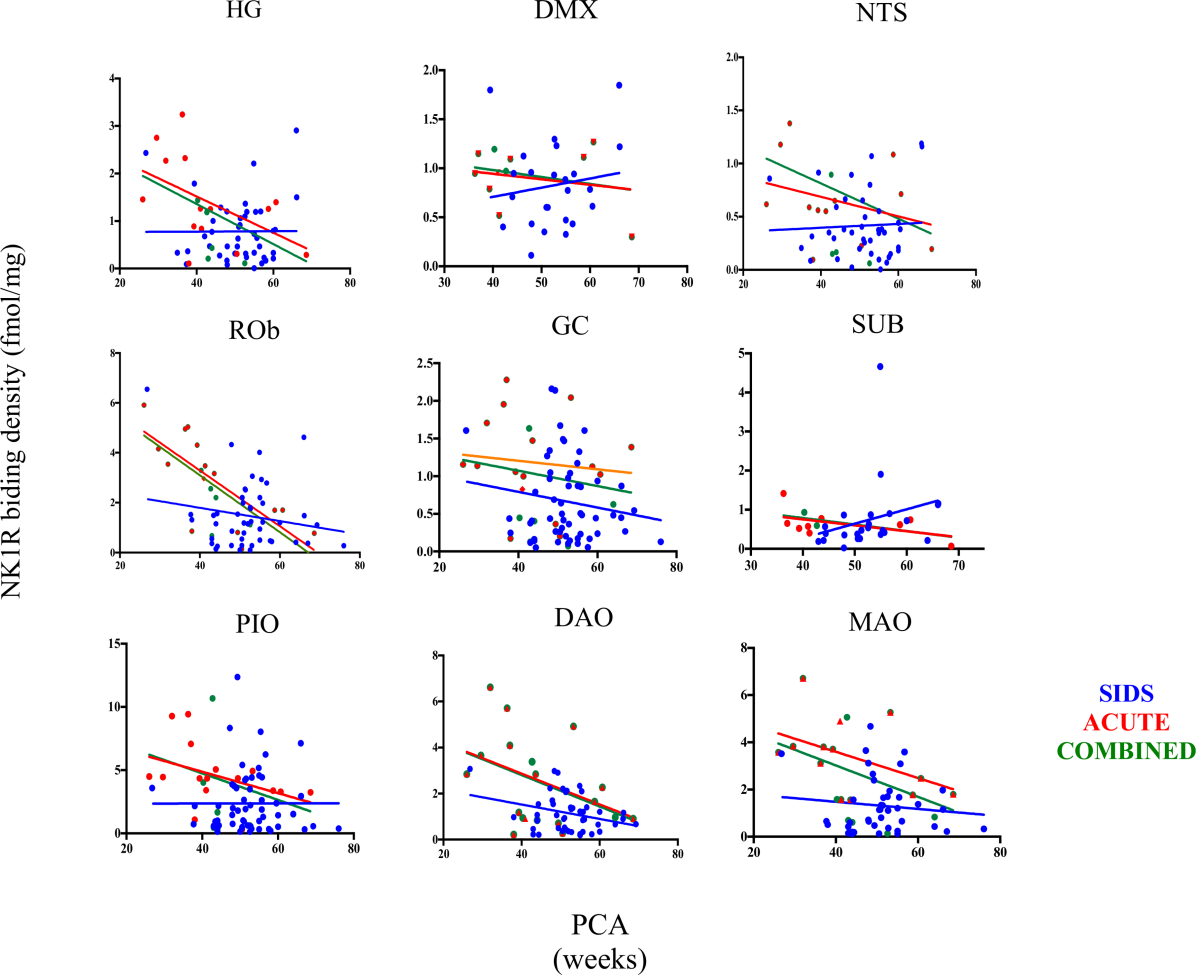
NK1R binding by PCA across diagnoses in multiple medullary nuclei.

Fig 4 shows analysis of binding by PCA across diagnoses in select medullary nuclei and reveals a clear trend for binding to decrease with age in all nuclei displayed in non-SIDS controls, although a statistically significant reduction in binding was observed only in the ROb (acute controls p = 0.009, combined controls p = 0.04). In contrast, no consistent age-related pattern in binding was observed in SIDS cases, with a trend for binding to *increase* with age observed in several nuclei including the DMX, HG, NTS and SUB, while significant age-related *reductions* in NK1R binding were observed in the DAO (p = <0.001) and MAO (p = 0.02) (Fig 4). Notably, a significant age vs. diagnosis interaction was observed in the HG (p = 0.03) and DMX (p = <0.001), where binding decreased with age in acute controls but not in SIDS cases (Fig 4).

**Fig 5:**
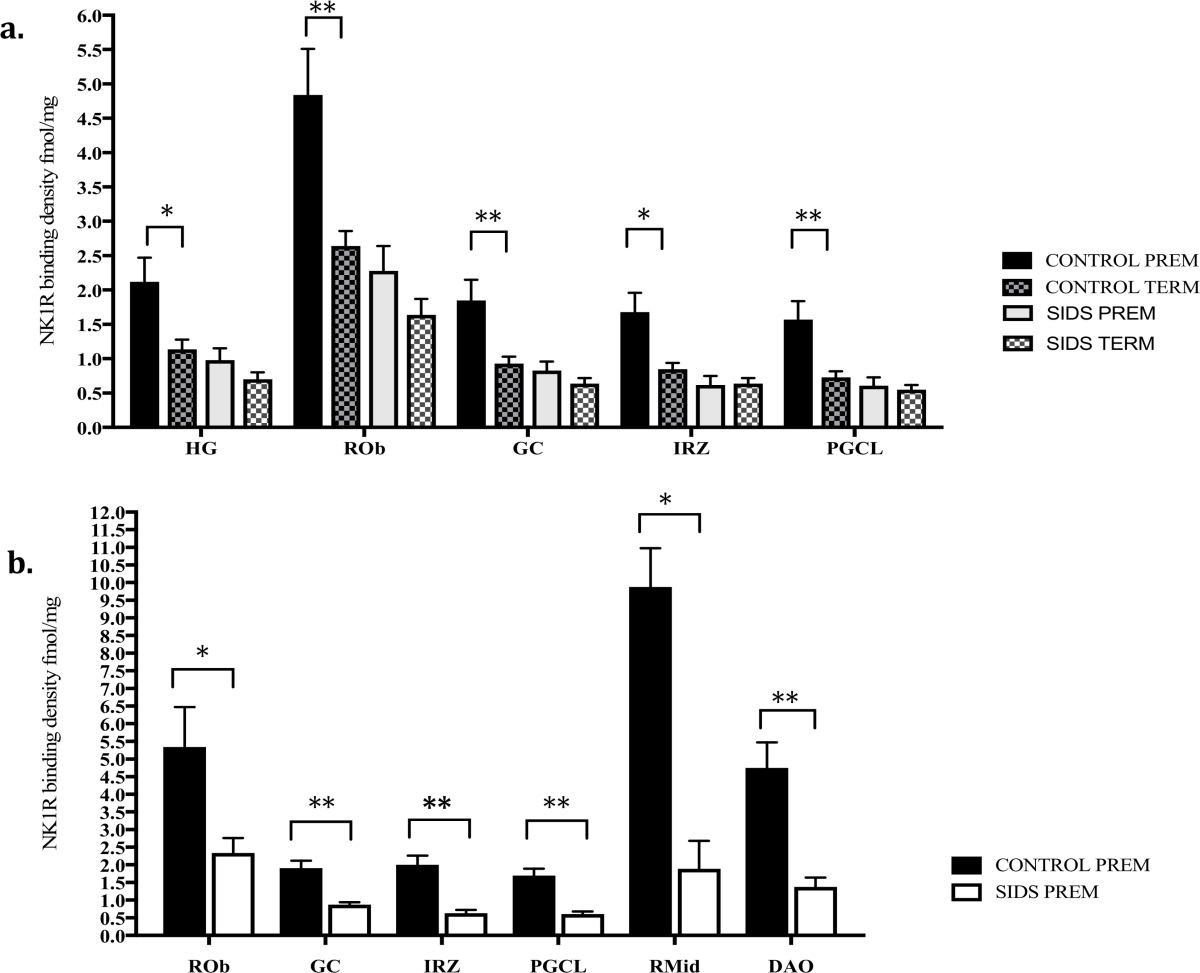
a. Significant effect of prematurity on NK1R binding in key medullary nuclei in term and premature non-SIDS controls vs. no effect in term compared to premature SIDS cases. b. Signficaint differences in NK1R binding in premature SIDS cases compared to premature controls in key medullary nuclei. *p = <0.05, **p = <0.01.

To determine the influence of prematurity status on observations, the density of NK1R sites in premature infants (defined as infants with gestational age <36 weeks) was compared to term infants (gestational age ≥ 36 weeks). Although only n = 3 premature infants (n = 2 acute controls, n = 1 hypoxic) Fig 5 shows the effect of prematurity on NK1R binding in key medullary nuclei analyzed. A striking trend for increased binding (>50% higher in every nucleus analyzed) observed in premature control infants compared to term control infants, with significant increases present in the HG (p = 0.02), ROb (p = 0.006), GC (p = 0.008), IRZ (p = 0.01) and PGCL (p = 0.007) (Fig 5A). In contrast, no significant differences in NK1R binding were observed between premature and term SIDS infants in any of the nuclei analyzed (Fig 5A). Moreover, NK1R binding was observed to be significantly lower in premature SIDS infants in the ROb (p = 0.04), GC (p = 0.001), IRZ (p = <0.001), PGCL (p = <0.001), RMid (p = 0.04) and DAO (p = 0.003) compared to premature control infants (Fig 5B).

### Analysis of [^125^I] BH-SP binding to NK1R adjusted by sex

**Fig 6:**
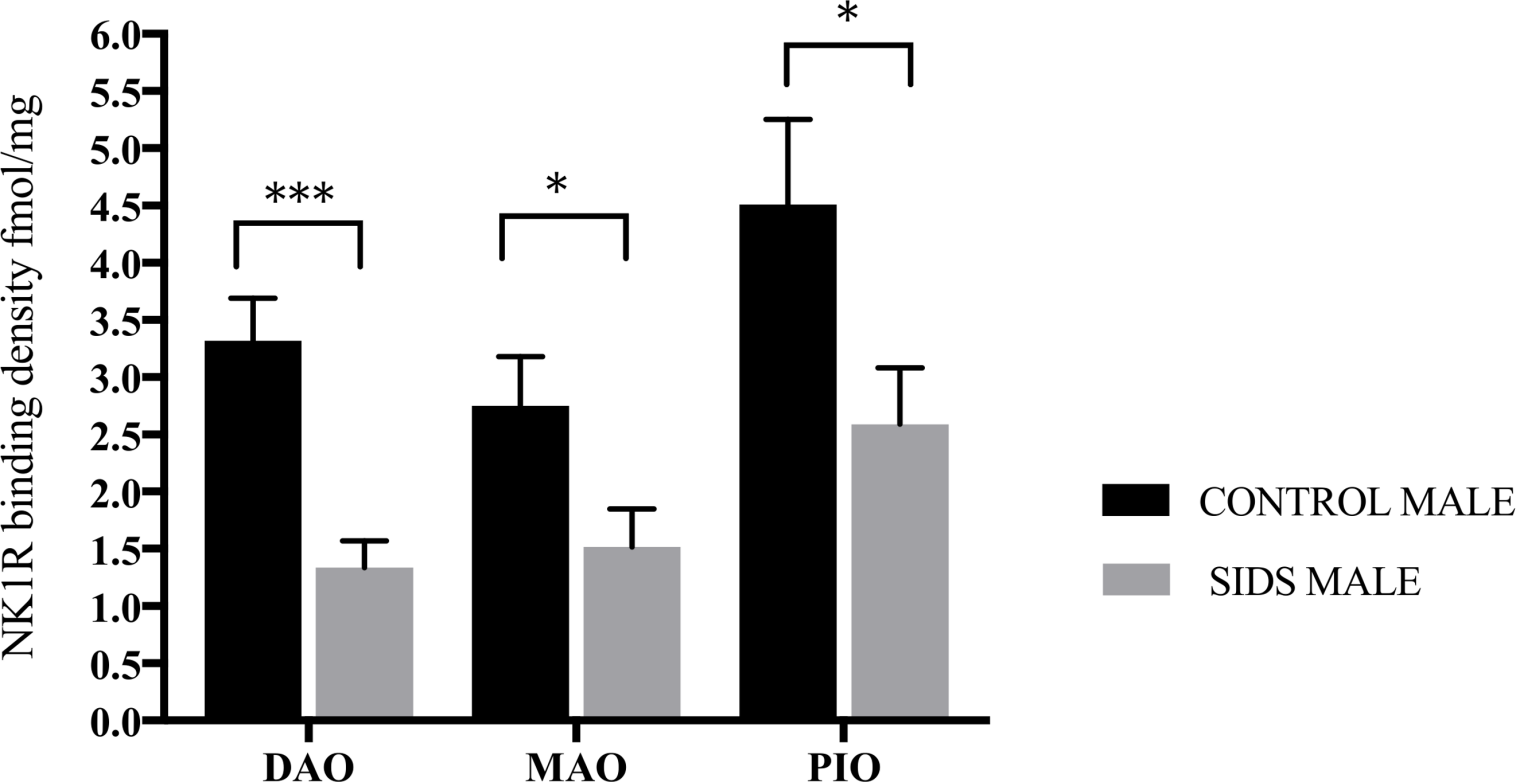
Marked sex effect on NK1R binding observed in the IO nuclei in male SIDS cases. *p = <0.05, ***p<0.001.

Analysis of binding by sex revealed an overall trend for higher binding in male compared to female infants in both acute and combined control cohorts. In acute controls, significantly higher binding was observed in males in the ROb (p = 0.02) and DAO (p = 0.02), and borderline significance in PGCL (p = 0.09) and GC (p = 0.07). In combined controls, NK1R binding was significantly higher in males in the DAO (p = 0.02), with borderline significance in ROb (p = 0.06) and GC (p = 0.09). In contrast, no differences in NK1R binding were observed between male and female SIDS cases in any of the nuclei analyzed. Furthermore, compared to controls a trend for NK1R binding in male SIDS cases to be reduced across all nuclei analyzed was observed, with Fig 6 showing significant reductions in all three IO component nuclei (DAO p = <0.001, MAO p = 0.03, PIO p = 0.04) in male SIDS cases when compared to male combined controls. In contrast there were no significant differences in binding observed between female SIDS and female acute or combined controls.

The small number of control cases precluded investigation of the potential interaction between prematurity and sex on NK1R binding, however such analysis in SIDS cases revealed a trend for higher binding in several nuclei in premature male SIDS cases compared to term male SIDS cases although a statistically significant increase was only observed in the HG (p = 0.005). In contrast, no trend for increased binding and no statistically significant differences in binding were observed in premature female SIDS infants and to term female SIDS infants in any of the nuclei analyzed. Comparison of binding in premature male and premature female SIDS cases also revealed no significant differences.

## Discussion

SP and the NK1R have been shown to play an integral role in the modulation of homeostatic function in the medulla, including regulation of respiratory rhythm generation [14–16], integration of cardiovascular control [17], modulation of the baroreceptor reflex [18] and mediation of the chemoreceptor reflex in response to hypoxia [19, 20, 28]. In this study, we observed abnormalities related to development and sex in NK1R binding within multiple nuclei of the medullary homeostatic network in SIDS cases incuding absolute reductions in binding in the NTS and and all sub-divisions of IO nuclei (PIO, MAO, DAO). The NTS houses the primary relay station for brainstem transmission of important respiratory and cardiovascular reflexes and is enriched with a high density of SP containing axon terminals [29, 30]. Evidence from animal studies indicates a functional role for SP in the NTS as a central integrator of cardiovascular control [17], modulator of baroreceptor reflex sensitivity [18] and a primary excitatory mediator of the chemoreceptor reflex in response to hypoxia [19, 20]. In rodent models, activation of NK1R by SP in the NTS stimulates respiration, while loss of NK1R reduces the respiratory response, severely impairing the chemoreceptor reflex and selective lesion of NK1R expressing neurons in the NTS blunts cardiovascular reflexes [31, 32]. These observations support the idea that the abnormal expression of NK1R in the medulla, as observed in this study, may result in dysfunction of critical cardiorespiratory reflexes in response to harmful stimuli such as hypoxia.

In contrast to the NTS, the IO nuclei are generally not considered to play role in the regulation and coordination of homeostatic function. Rather the IO is a pre-cerebellar relay network providing the climbing fibers to Purkinje cells in the cerebellar cortex and a central site for the integration of motor and sensory information [33, 34] [35]. Previous studies have associated neurotransmitter deficits [22, 23], significantly reduced neuron density [36] and substantial reactive gliosis [37] within the IO with a subset of SIDS deaths and the IO, through its projections to the cerebellum, has been identified to influence upper airway and respiratory muscle regulation and blood pressure control in response to hypotensive challenge by coordinating and synchronizing somatomotor, respiratory and autonomic actions [38, 39]. In addition, afferent input to the cerebellum via the IO or climbing purkinje fibers from the olivary nuclei have been implicated in the failure of cerebellar mechanisms to produce adequate somatomotor response (i.e. head lift/tilt, respiratory muscle activity) to overcome cardiorespiratory challenges during sleep in SIDS [40]. The density and distribution of SP positive immunoreactivity within the IO has indicate a modulatory role for the neuropeptide in olivary neuron output and activity [41]. Taking the above observations together, we suggest that the significantly abnormal expression of NK1R in both the IO and NTS in SIDS cases may contribute to an inability of a SIDS infant to execute appropriate motor responses in order to respond to life threatening challenges during sleep, which may underlie the pathogenesis of SIDS in conjunction with other neurotransmitter abnormalities within the same or associated medullary nuclei.

A striking feature of the abnormalities in NK1R expression in SIDS cases observed in this study is their association with prematurity and male sex both of which are recognized risk factors for SIDS. In controls, premature infants had significantly higher binding than in term infants and binding was observed to decrease with age across all nuclei. This pattern is consistent with the developmental expression of NK1R, with animal studies reporting peak NK1R density at birth, which decreased over the course of development to reach adult levels of expression [27, 42]. In contrast, no significant differences in binding were observed between premature and term SIDS infants and no consistent age-related reduction in NK1R expression was observed with development. Indeed, a paradoxical trend for binding to increase with age was observed in the HG and DMX in SIDS cases with a significant age versus diagnosis interaction observed in these nuclei. Moreover, NK1R binding in premature SIDS infants was significantly lower than in premature controls infants in the ROB, GC and PGCL, which are recognized as key autonomic and respiratory control nuclei within the medullary homeostatic network. Significant alterations in NK1R binding within the PGCL nuclei is of particular interest, given the PGCL is the proposed location of the putative human homologue of the Prebotzinger complex (PBC). Depletion of SP within the PBC has been shown in animal studies to offset ventilatory rhythm generation in neonates [43] and lesioning of NK1R expressing neurons within the PBC results in profoundly abnormal respiratory patterns [14, 44]. Animal models have shown that juveniles are more sensitive to SP and its modulation of respiratory activity is more important during the early postnatal period particularly when the juvenile is challenged by hypoxia [45, 46]. This indicates that reduced NK1R expression in SIDS infants and premature SIDS infants in particular places them at increased risk of respiratory failure and this observation may at least partially explain the increased risk of SIDS associated with prematurity. Moreover, these observations indicate that the pathogenesis of altered NK1R expression in SIDS originates during gestation and further supports the idea that SIDS is a developmental disorder with a prenatal etiology.

In addition to prematurity, male sex was significantly associated with reduced NK1R binding in SIDS cases. In control infants, binding in male infants was observed to be significantly higher than in females, in contrast no such difference was observed between male and female SIDS cases. Moreover, binding in male but not female SIDS cases was observed to be significantly lower compared to male and female controls respectively. These observations indicate that NK1R binding is reduced in male but not female SIDS infants and that the reductions in binding observed in the SIDS cohort as whole are driven, along with prematurity, by male infants. Notably, males have been shown to normally have a reduced ventilatory response to hypoxia and a longer post hypoxic recovery compared to females in experimental models [47, 48]. Taken together, these observations suggest that premature male infants are a population that is at an elevated risk of SIDS.

### Limitations

A potential limitation to this study is the relatively small sample size of control cases for each diagnoses and in particular the small cohort of premature control cases (n = 2 acute, n = 1 hypoxic) available for analysis. Infant control cases are especially difficult to obtain due to the rarity of deaths in the age group (<12 months) from causes of death other than SIDS. However despite the small sample size, we found highly significant differences (>50% higher in every nucleus analyzed) in NK1R binding in premature compared to term combined controls, compared to no significant differences observed in premature compared to term SIDS cases. Another potential limitation is that in some instances we observed trends for differences between SIDS and control cases that were not statistically significant due to a lack of statistical power. Future analysis to clarify the effect of prematurity and trends observed in NK1R binding requires a larger dataset of controls.

## Conclusion

In summary this study has identified a subset of SIDS infants with a significant developmental abnormality of the SP/NK1R system, with altered NK1R binding in multiple nuclei intimately related to cardiorespiratory function and autonomic control within the medulla oblongata. Our observations were influenced by prematurity and male sex, which may further explain the increased risk of SIDS in premature and male infants. Collectively these observations suggest that abnormal SP neurotransmission within the medulla could result in dysfunction of critical cardiorespiratory reflexes in response to harmful stimuli such as hypoxia and may contribute to an inability of an infant to execute appropriate motor responses to life threatening challenges during sleep. Furthermore our observations support the concept that abnormalities in a multi-neurotransmitter network underlie the pathogenesis of a subset of SIDS infants and that SIDS is a complex developmental disorder with a prenatal etiology.

## Supporting information

S1 Table Analysis of the effect of prematurity status on mean total NK1R binding (fmol/mg) across nuclei and diagnoses. Significant effects of prematurity were observed in controls in multiple nuclei, with a trend for increased binding in premature compared with term cases across nuclei in acute and combined controls. No significant effects of prematurity were observed in the SIDS cohort. Significance at level p = <0.05.
(DOCX)

S2 Table Mean total NK1R binding (fmol/mg) by sex across medullary nuclei and diagnoses. Binding was significantly higher in multiple nuclei in control males vs. control females, with a trend for binding to be higher in males across nuclei analyzed. In SIDS however, there were no significant differences in binding between male sand females, with the exception of trend for binding to be higher in PGCL nuclei. Significance at level p = <0.05.
(DOCX)

S3 Table NK1R binding density (fmol/mg) across 13 selected medullary nuclei in SIDS and non-SIDS control infant dataset. (DOCX)
