# Supplementary material for: Abnormalities in substance P neurokinin-1 receptor binding in key brainstem nuclei in sudden infant death syndrome related to prematurity and sex
Source: PLoS One. 2017 Sep 20;12(9):e0184958. doi: 10.1371/journal.pone.0184958 (PMC5607183; doi:10.1371/journal.pone.0184958)
Supplement: S1 Table — Significant effects of prematurity were observed in controls in multiple nuclei, with a trend for increased binding in premature compared with term cases across nuclei in acute and combined controls. No significant effects of prematurity were observed in the SIDS cohort. Significance at level p = <0.05. (DOCX) [file pone.0184958.s001.docx]

|  | **SIDS** | | | **Acute Controls** | | | **Combined Controls** | | |
| --- | --- | --- | --- | --- | --- | --- | --- | --- | --- |
|  | **Premature**  **mean (±SE)**  **N=12** | **Term**  **mean (±SE)**  **N=43** | **P**  **value** | **Premature**  **mean (±SE)**  **N=2** | **Term**  **mean (±SE)**  **N=13** | **P**  **value** | **Premature**  **mean (±SE)**  **N=3** | **Term**  **mean (±SE)**  **N=18** | **P**  **value** |
| **HG** | 0.98 (0.17) | 0.7 (0.1) | 0.17 | 2.74 (0.41) | 1.16 (0.16) | 0.005 | 2.12 (0.35) | 1.14 (0.14) | 0.02 |
| **DMX** | 1.08 (0.26) | 0.86 (0.09) | 0.43 | 0.97 (0.25) | 0.89 (0.14) | 0.78 | 0.98 (0.23) | 0.9 (0.12) | 0.76 |
| **NTS** | 0.52 (0.09) | 0.39 (0.05) | 0.26 | 0.05 (1.13) | 0.8 (0.34) | 0.53 | 0.35 (0.59) | 0.77 (0.21) | 0.5 |
| **SUB** | 0.68 (0.72) | 0.89 (0.24) | 0.79 | 0.93 (0.25) | 0.6 (0.14) | 0.3 | 0.95 (0.23) | 0.59 (0.12) | 0.22 |
| **Rob** | 2.28 (0.36) | 1.64 (0.23) | 0.14 | 4.55 (0.66) | 2.62 (0.24) | 0.02 | 4.84 (0.67) | 2.64 (0.22) | 0.006 |
| **GC** | 0.83 (0.13) | 0.64 (0.08) | 0.24 | 2 (0.33) | 0.91 (0.12) | 0.008 | 1.85 (0.3) | 0.93 (0.1) | 0.008 |
| **IRZ** | 0.62 (0.13) | 0.64 (0.08) | 0.89 | 1.79 (0.33) | 0.84 (0.12) | 0.02 | 1.68 (0.28) | 0.85 (0.09) | 0.01 |
| **PGCL** | 0.61 (0.12) | 0.55 (0.07) | 0.69 | 1.66 (0.32) | 0.72 (0.11) | 0.01 | 1.57 (0.27) | 0.73 (0.09) | 0.007 |
| **RMID** | 3.13 (1.23) | 3.73 (0.4) | 0.65 | 7.89 (1.11) | 3.93 (0.61) | 0.03 | 7.68 (1.27) | 4.44 (0.65) | 0.07 |
| **DAO** | 1.33 (0.21) | 1.13 (0.17) | 0.43 | 5.29 (1.36) | 2.26 (0.48) | 0.06 | 5.04 (1.23) | 2.37 (0.42) | 0.06 |
| **PIO** | 2.81 (0.63) | 2.08 (0.39) | 0.31 | 8.3 (1.47) | 4 (0.53) | 0.02 | 7.27 (1.76) | 4.05 (0.57) | 0.09 |
| **MAO** | 1.62 (0.36) | 1.2 (0.24) | 0.34 | 4.49 (1.46) | 3.67 (0.59) | 0.59 | 2.81 (1) | 2.7 (0.45) | 0.92 |
| **ARC** | 0.42 (0.21) | 0.44 (0.12) | 0.94 | 0.1 (0.77) | 0.29 (0.37) | 0.84 | 0.14 (0.52) | 0.33 (0.21) | 0.76 |
|  |  |  |  |  |  |  |  |  |  |

**S1 Table. Analysis of the effect of prematurity status on mean total NK1R binding (fmol/mg) across nuclei and diagnoses.** Significant effects of prematurity were observed in controls in multiple nuclei, with a trend for increased binding in premature compared with term cases across nuclei in acute and combined controls. No significant effects of prematurity were observed in the SIDS cohort. Significance at level p=<0.05.
