# Supplementary material for: Abnormalities in substance P neurokinin-1 receptor binding in key brainstem nuclei in sudden infant death syndrome related to prematurity and sex
Source: PLoS One. 2017 Sep 20;12(9):e0184958. doi: 10.1371/journal.pone.0184958 (PMC5607183; doi:10.1371/journal.pone.0184958)
Supplement: S2 Table — Binding was significantly higher in multiple nuclei in control males vs. control females, with a trend for binding to be higher in males across nuclei analyzed. In SIDS however, there were no significant differences in binding between male sand females, with the exception of trend for binding to be higher in PGCL nuclei. Significance at level p = <0.05. (DOCX) [file pone.0184958.s002.docx]

|  | SIDS | | | Acute Controls | | | Combined Controls | | |  |
| --- | --- | --- | --- | --- | --- | --- | --- | --- | --- | --- |
|  | Male  mean (±SE)  N=33 | Female  mean (S±E)  N=22 | P  value | Male  mean (±SE)  N=8 | Female  mean (±SE)  N=7 | P  value | Male  mean (S±E)  N=12 | Female  mean (±SE)  N=9 | P  value |  |
| HG | 0.69 (0.12) | 0.92 (0.13) | 0.17 | 1.54 (0.29) | 0.91 (0.37) | 0.21 | 1.38 (0.19) | 0.99 (0.26) | 0.24 |  |
| DMX | 0.9 (0.15) | 0.86 (0.12) | 0.85 | 0.69 (0.18) | 1.04 (0.13) | 0.2 | 0.76 (0.14) | 1.04 (0.12) | 0.22 |  |
| NTS | 0.43 (0.06) | 0.43 (0.07) | 0.99 | 0.53 (0.44) | 1.13 (0.55) | 0.44 | 0.62 (0.24) | 0.95 (0.33) | 0.42 |  |
| SUB | 0.54 (0.36) | 1.13 (0.32) | 0.27 | 0.63 (0.22) | 0.71 (0.17) | 0.81 | 0.61 (0.18) | 0.72 (0.15) | 0.68 |  |
| Rob | 1.82 (0.25) | 1.85 (0.28) | 0.93 | 3.36 (0.29) | 2.06 (0.34) | 0.02 | 3.15 (0.29) | 2.19 (0.37) | 0.06 |  |
| GC | 0.77 (0.08) | 0.57 (0.1) | 0.1 | 1.22 (0.18) | 0.65 (0.22) | 0.07 | 1.12 (0.13) | 0.74 (0.17) | 0.09 |  |
| IRZ | 0.7 (0.08) | 0.53 (0.1) | 0.17 | 1.1 (0.18) | 0.63 (0.21) | 0.12 | 1 (0.13) | 0.73 (0.17) | 0.21 |  |
| PGCL | 0.64 (0.07) | 0.44 (0.09) | 0.07 | 0.99 (0.17) | 0.5 (0.2) | 0.09 | 0.87 (0.12) | 0.6 (0.16) | 0.2 |  |
| RMID | 3.84 (0.51) | 3.41 (0.66) | 0.62 | 5.73 (1.1) | 4.12 (1.1) | 0.38 | 5.96 (0.91) | 4.15 (1.04) | 0.27 |  |
| DAO | 1.24 (0.14) | 1.07 (0.18) | 0.37 | 3.55 (0.57) | 1.12 (0.66) | 0.02 | 3.49 (0.5) | 1.33 (0.57) | 0.02 |  |
| PIO | 2.35 (0.4) | 2.29 (0.5) | 0.92 | 4.98 (0.83) | 3.36 (1) | 0.24 | 4.86 (0.69) | 3.13 (0.91) | 0.13 |  |
| MAO | 1.45 (0.23) | 1.13 (0.28) | 0.36 | 4.11 (0.66) | 2.91 (1) | 0.34 | 2.97 (0.45) | 1.95 (0.73) | 0.23 |  |
| ARC | 0.56 (0.09) | 0.23 (0.13) | 0.08 | -0.45 (0.01) | 0.46 (0) | 0.006 | -0.4 (0.02) | 0.51 (0.01) | <0.001 |  |

**S2 Table.** **Mean total** **NK1R binding by sex across medullary nuclei and diagnoses.** Binding was significantly higher in multiple nuclei in control males vs. control females, with a trend for binding to be higher in males across nuclei analyzed. In SIDS however, there were no significant differences in binding between male sand females, with the exception of trend for binding to be higher in PGCL nuclei. Significance at level p=<0.05
