# Supplementary material for: Abnormalities in substance P neurokinin-1 receptor binding in key brainstem nuclei in sudden infant death syndrome related to prematurity and sex
Source: PLoS One. 2017 Sep 20;12(9):e0184958. doi: 10.1371/journal.pone.0184958 (PMC5607183; doi:10.1371/journal.pone.0184958)
Supplement: S3 Table — (DOCX) [file pone.0184958.s003.docx]

|  |  | **NK1R binding density (fmol/mg) across 13 selected medullary nuclei in SIDS and non-SIDS control infant dataset** | | | | | | | | | | | | |
| --- | --- | --- | --- | --- | --- | --- | --- | --- | --- | --- | --- | --- | --- | --- |
| **Case#** | **Diagnosis** | **HG** | **DMx** | **NTS** | **SUB** | **Rob** | **GC** | **IRZ** | **PGCL** | **R.Mid** | **DAO** | **PIO** | **MAO** | **Arc** |
| 1 | SIDS | 0.634 | NA | 0.653 | NA | 1.992 | 0.641 | 0.680 | 0.656 | NA | 0.900 | 1.019 | 1.151 | NA |
| 2 | SIDS | 2.432 | NA | 0.858 | NA | 6.549 | 1.607 | 1.364 | 1.106 | NA | 3.071 | 3.576 | 3.516 | NA |
| 3 | SIDS | 0.470 | NA | 0.296 | NA | 1.494 | 0.408 | 0.449 | 0.379 | NA | NA | 0.921 | 0.599 | NA |
| 4 | SIDS | 1.060 | NA | 0.496 | NA | 2.517 | 0.416 | 0.480 | 0.349 | NA | 0.432 | 1.914 | 1.323 | NA |
| 5 | SIDS | 0.365 | NA | 0.313 | NA | 1.529 | 0.437 | 0.489 | 0.393 | NA | NA | 0.719 | 0.652 | NA |
| 6 | SIDS | 0.315 | NA | 0.275 | NA | 1.240 | 0.364 | 0.383 | 0.329 | NA | 0.331 | 0.538 | 0.364 | NA |
| 7 | SIDS | 0.319 | NA | 0.198 | NA | 0.107 | 0.126 | 0.168 | 0.116 | NA | NA | 0.141 | NA | NA |
| 8 | SIDS | 0.648 | NA | 0.382 | NA | 0.282 | 0.262 | 0.31 | 0.264 | NA | 0.375 | 0.421 | NA | 0.301 |
| 9 | SIDS | 0.01 | NA | 0.096 | NA | 0.158 | 0.142 | 0.148 | 0.097 | NA | NA | 0.128 | NA | NA |
| 10 | SIDS | 0.111 | NA | 0.067 | NA | 1.978 | 0.231 | 0.209 | 0.169 | NA | 0.334 | 1.881 | NA | NA |
| 11 | SIDS | 0.334 | NA | 0.205 | NA | 0.373 | 0.267 | 0.329 | 0.241 | NA | NA | 0.3 | 0.219 | NA |
| 12 | SIDS | 0.231 | NA | 0.119 | NA | 0.945 | 0.172 | 0.128 | 0.103 | NA | 0.239 | 0.973 | 0.237 | NA |
| 13 | SIDS | 0.239 | NA | 0.151 | NA | 0.473 | 0.055 | 0.05 | 0.054 | NA | NA | 0.327 | NA | NA |
| 14 | SIDS | 0.17 | NA | 0.148 | NA | 1.543 | 0.265 | 0.225 | 0.265 | NA | 0.503 | 1.791 | 0.478 | 0.443 |
| 15 | SIDS | 0.212 | NA | 0.182 | NA | 0.277 | 0.145 | 0.162 | 0.136 | NA | NA | 0.348 | NA | 0.132 |
| 16 | SIDS | NA | NA | NA | NA | 0.442 | 0.169 | 0.135 | 0.136 | NA | 0.239 | 0.192 | NA | NA |
| 17 | SIDS | 0.094 | NA | 0.085 | NA | 0.1 | 0.104 | 0.12 | 0.074 | NA | NA | 0.125 | NA | NA |
| 18 | SIDS | 0.274 | NA | 0.379 | NA | 0.274 | 0.129 | 0.202 | 0.136 | NA | NA | 0.374 | 0.332 | NA |
| 19 | SIDS | 0.331 | NA | 0.209 | NA | 1.314 | 0.246 | 0.197 | 0.258 | NA | 0.977 | 2.154 | 0.515 | NA |
| 20 | SIDS | 0.079 | NA | 0.023 | NA | 0.551 | 0.119 | 0.113 | 0.078 | NA | 0.224 | 0.526 | 0.148 | NA |
| 21 | SIDS | 0.793716667 | 0.784916667 | 0.441116667 | 0.395216667 | 0.249666667 | 0.0526 | 0.056125 | 0.037225 | 0.1585 | 0.21405 | 0.612658333 | 0.187120833 | NA |
| 22 | SIDS | 0.33515 | 0.32565 | 0.3456 | 0.19075 | 0.3658 | 0.37765 | 0.41985 | 0.27055 | 1.0178 | 0.462475 | 0.585625 | 0.4294 | 0.37905 |
| 23 | SIDS | 0.4681 | 0.43295 | 0.34985 | 0.217 | 0.1416 | 0.1638 | 0.5068 | 0.4705 | 0.5851 | 0.828475 | 0.99525 | 0.116625 | 0.1354 |
| 24 | SIDS | 1.7877 | 1.79865 | 0.91435 | 0.7212 | 1.2036 | 0.93715 | 1.08395 | 0.8237 | 0.4513 | 1.2072 | 1.4282125 | 1.372475 | 1.2194 |
| 25 | SIDS | NA | NA | NA | NA | NA | 0.23668 | 0.23293 | 0.17538 | 0.58208 | 0.34748 | 0.51353 | NA | NA |
| 26 | SIDS | 0.4681 | 0.43295 | 0.34985 | 0.217 | 0.409 | 0.4836 | 0.50375 | 0.41335 | 0.9232 | 0.668 | 1.52335 | 0.4339 | 0.6852 |
| 27 | SIDS | 0.7738 | 0.70865 | 0.5911 | 0.37305 | 0.5004 | 0.104125 | 0.157175 | 0.104425 | 0.1824 | 0.191675 | 0.3119 | 0.12825 | NA |
| 28 | SIDS | NA | NA | NA | NA | NA | 0.85867 | 0.73082 | 0.76927 | 1.75362 | 1.20522 | 4.430605 | NA | NA |
| 29 | SIDS | NA | NA | NA | NA | NA | 0.4429 | 0.39805 | 0.27975 | 2.2023 | 0.6566 | 2.374525 | NA | NA |
| 30 | SIDS | 2.2104 | 0.8864 | 0.37405 | 4.66255 | 4.018 | 1.1727 | 1.18485 | 0.97605 | 5.58585 | 2.3347 | 5.1601375 | NA | NA |
| 31 | SIDS | 1.3634 | 1.2964 | 0.7995 | 0.63465 | 1.768 | 0.87075 | 0.75045 | 0.48435 | 3.1602 | 1.32855 | 2.2272875 | 1.1143 | NA |
| 32 | SIDS | 1.0029 | 0.94755 | 0.099 | 0.57125 | 1.565 | 0.7917 | 0.731 | 0.7073 | 2.5792 | 1.53895 | 2.167125 | 1.564775 | 0.78025 |
| 33 | SIDS | 2.90615 | 1.84825 | 1.18755 | 1.12835 | 4.6232 | 0.4482 | 0.6005 | 0.460525 | 1.4725 | 0.900725 | 7.1164125 | 1.9748375 | NA |
| 34 | SIDS | 0.88075 | 0.60135 | 0.2849 | 0.2656 | 0.7864 | 0.49995 | 0.4475 | 0.5393 | 1.2294 | 0.9379 | 2.634475 | 1.33355 | NA |
| 35 | SIDS | 0.72915 | 0.47175 | 0.5516 | 1.90795 | 2.2017 | 0.87135 | 0.6177 | 0.63995 | 4.232 | 1.223325 | 4.565425 | 1.208175 | NA |
| 36 | SIDS | 1.19365 | 0.7743 | 0.00285 | 0.4691 | 2.9295 | 1.32505 | 1.2563 | 1.2004 | 5.0851 | 2.10095 | 8.028 | 1.67035 | NA |
| 37 | SIDS | NA | NA | NA | NA | NA | 0.48395 | 0.38705 | 0.377 | 0.8527 | 0.8764 | 1.293525 | 0.65945 | NA |
| 38 | SIDS | 1.49905 | 1.2191 | 1.1614 | 1.16205 | 1.4787 | 0.8703 | 0.926 | 0.78925 | 3.5897 | 1.1673 | 2.93455 | 1.151775 | 0.11615 |
| 39 | SIDS | NA | NA | NA | NA | NA | 1.46895 | 1.1804 | 1.24845 | 5.4904 | 2.12515 | 4.315725 | 1.65205 | 1.063 |
| 40 | SIDS | NA | NA | NA | NA | NA | 2.1391 | 2.06515 | 1.76945 | 5.07755 | 2.9097 | 12.355475 | 2.3948 | NA |
| 41 | SIDS | 1.1941 | 1.23 | 1.06995 | 0.87505 | 3.0639 | 1.04025 | 1.1267 | 1.0708 | 3.97705 | 1.3685 | 4.4077375 | 1.9353875 | 0.7585875 |
| 42 | SIDS | 0.9168 | 0.6004 | 0.256275 | 0.47935 | 2.5445 | 1.4901 | 1.3966 | 1.05835 | 6.5571 | 2.0665 | 4.210325 | NA | NA |
| 43 | SIDS | NA | NA | NA | NA | NA | 1.2681 | 0.4296 | 0.89525 | 4.8492 | 2.23455 | 8.3211 | 3.6497 | NA |
| 44 | SIDS | 0.6457 | NA | NA | 0.4193 | 1.2239 | 0.5046 | 0.4434 | 0.4405 | 2.7361 | 0.85105 | 2.615125 | 3.09005 | NA |
| 45 | SIDS | 1.20195 | 0.95825 | 0.89425 | 0.8671 | 4.3353 | 1.34095 | 1.1604 | 1.07875 | 4.6363 | 1.694975 | 3.8639285 | 3.117675 | 0.8863 |
| 46 | SIDS | 0.46525 | 0.35085 | 0.4055 | 0.26355 | 2.20335 | 1.67165 | 1.7751 | 1.44195 | 3.9929 | 2.21275 | 5.3961 | 1.1446 | NA |
| 47 | SIDS | 1.2014 | 0.94175 |  | 0.90265 | 2.7914 | 1.60685 | 1.553 | 1.2726 | 4.4881 | 1.40435 | 6.2289875 | 3.59085 | NA |
| 48 | SIDS | 0.6787 | 0.40185 | 0.3496 | 0.2169 | 1.1478 | 1.13245 | 0.70765 | 0.80485 | 2.837 | 2.061625 | 5.423175 | 2.76465 | NA |
| 49 | SIDS | NA | NA | NA | NA | NA | 2.2064 | 2.04855 | 2.1115 | 5.49 | 2.3424 | 5.69765 | 0.71135 | NA |
| 50 | SIDS | 0.169825 | 0.1117 | NA | 0.0251 | 0.2851 | 0.9668 | 0.92575 | 0.9216 | 1.0733 | 1.7038 | 1.445675 | 0.7148 | NA |
| 51 | SIDS | NA | NA | NA | NA | NA | 0.6884 | 0.82155 | 0.6209 | 2.4052 | 1.06135 | 3.574575 | 2.6552 | NA |
| 52 | SIDS | 1.28635 | 1.12545 | 0.66358 | 0.7326 | 1.4202 | 1.0894 | 0.32825 | 0.4038 | 2.2482 | 0.6409 | 1.626225 | 0.858655556 | NA |
| 53 | SIDS | 0.8171 | 0.6121 | 0.3821 | 0.3555 | 0.914 | 0.662375 | 0.52895 | 0.450325 | 2.6582 | 0.87945 | 1.6558375 | 0.5515875 | NA |
| 54 | SIDS | NA | NA | NA | NA | NA | 2.2429 | 1.87095 | 1.6627 | 11.3282 | 3.4982 | 9.108525 | 4.8567 | NA |
| 55 | SIDS | 1.095 | 0.932 | NA | 0.54335 | 1.8107 | 0.9778 | 0.9927 | 0.90195 | 2.281 | 1.3982 | 3.4939125 | 1.7006125 | 0.2203 |
| 56 | Acute Control | 2.271 | NA | 1.377 | NA | 3.543 | 1.708 | 1.502 | 1.351 | NA | 6.620 | 9.271 | 6.712 | NA |
| 57 | Acute Control | 2.753 | NA | 1.178 | NA | 4.158 | 1.139 | 1.044 | 0.933 | NA | 3.652 | 4.442 | 3.840 | NA |
| 58 | Acute Control | 1.457 | NA | 0.616 | NA | 5.915 | 1.155 | 1.144 | 0.818 | NA | 2.844 | 4.484 | 3.578 | NA |
| 59 | Acute Control | 0.31 | NA | 0.225 | NA | 0.141 | 0.205 | 0.263 | 0.249 | NA | 0.28 | 0.289 | NA | 0.281 |
| 60 | Acute Control | NA | NA | NA | NA | 0.809 | 0.363 | 0.316 | 0.226 | NA | 0.706 | 4.347 | NA | NA |
| 61 | Acute Control | 0.108 | NA | 0.095 | NA | 0.861 | 0.173 | 0.117 | 0.143 | NA | 0.222 | 1.067 | NA | NA |
| 62 | Acute Control | 1.3966 | 1.2669 | 0.71265 | 0.7466 | 1.7079 | 1.0228 | 1.0347 | 1.06635 | 2.5765 | 2.26645 | 3.2807125 | 2.4786 | 0.2768 |
| 63 | Acute Control | 1.24945 | 1.0934 | 0.6488 | 0.7738 | 3.1686 | 1.47365 | 1.47415 | 1.37875 | 6.0757 | 2.84585 | 5.050275 | 1.56455 | NA |
| 64 | Acute Control | 2.32365 | 1.1482 | 0.58855 | 0.65435 | 5.0369 | 2.2802 | 1.66995 | 1.6912 | 8.6019 | 4.08945 | 7.0625125 | 3.810225 | 0.320625 |
| 65 | Acute Control | 3.24365 | 0.94635 | NA | 1.42015 | 4.9584 | 1.9547 | 2.14985 | 1.80665 | 7.4546 | 5.70975 | 9.4084125 | 3.1132625 | NA |
| 66 | Acute Control | NA | NA | NA | NA | NA | 2.0441 | 1.9113 | 1.69955 | 4.919 | 4.919 | 4.919 | 5.2709 | NA |
| 67 | Acute Control | 1.2574 | 1.11135 | 1.08455 | 0.6174 | 1.7028 | 1.127675 | 1.1042 | 1.0851 | 3.7862 | 1.65085 | 3.3714 | 1.78085 | 0.0146 |
| 68 | Acute Control | 0.88785 | 0.78745 | 0.56055 | 0.52275 | 4.3097 | 1.0599 | 1.13025 | 0.9313 | 4.58895 | 1.1785 | 4.3395 | 3.7123 | 0.38545 |
| 69 | Acute Control | 0.2898 | 0.299075 | 0.194675 | 0.0716 | 0.7744 | 1.3854 | 1.2007 | 1.07515 | 2.7075 | 0.905075 | 3.2318875 | 1.79725 | NA |
| 70 | Acute Control | 0.83845 | 0.51795 | 0.5508 | 0.4044 | 3.4811 | 0.9996 | 0.8127 | 0.7872 | 3.58705 | NA | 4.343175 | 1.580975 | 0.07435 |
| 71 | Hypoxia | 0.209 | NA | 0.15 | NA | 0.674 | 0.13 | 0.119 | 0.108 | NA | NA | 0.441 | 0.696 | NA |
| 72 | Hypoxia | 0.434 | NA | 0.165 | NA | 2.202 | 0.403 | 0.494 | 0.363 | NA | 0.844 | 1.661 | 0.611 | 0.36 |
| 73 | Chronic Control | 0.112 | NA | 0.061 | NA | 1.12 | 0.07 | 0.033 | 0.03 | NA | NA | 0.621 | 0.134 | NA |
| 74 | Chronic Control | NA | NA | NA | NA | NA | 0.62705 | 0.5883 | 0.4934 | 0.9904 | 0.96905 | 1.501025 | 0.8319 | NA |
| 75 | Chronic Control | 1.43655 | 1.1938 | 3.609185 | 0.93545 | 3.2984 | 0.4463 | 0.4809 | 0.32155 | NA | 0.942225 | 4.0112 | 1.5845 | NA |
| 76 | Chronic Control | 1.18485 | 0.9722 | 0.8947 | 0.5953 | 2.5606 | 1.6353 | 1.31005 | 0.95005 | 7.0314 | 3.38015 | 10.6636875 | 5.06065 | NA |

NA: binding measurement not available
